# Assessment of knowledge retention and the value of proctored ultrasound exams after the introduction of an emergency ultrasound curriculum

**DOI:** 10.1186/1472-6920-7-40

**Published:** 2007-10-30

**Authors:** Vicki E Noble, Bret P Nelson, A Nicholas Sutingco, Keith A Marill, Hilarie Cranmer

**Affiliations:** 1 Department of Emergency Medicine, Massachusetts General Hospital, 55 Fruit St., Boston, Massachusetts, USA; 2 Department of Emergency Medicine, Mount Sinai School of Medicine, 1190 Fifth Avenue, NY, New York, USA; 3 Department of Emergency Medicine, INOVA Fair Oaks Hospital, 3600 Joseph Siewick Drive, Fairfax, Virginia, USA; 4 Department of Emergency Medicine, Brigham and Women's Hospital, 32 Francis St., Boston, Massachusetts, USA

## Abstract

**Background:**

Optimal training required for proficiency in bedside ultrasound is unknown. In addition, the value of proctored training is often assumed but has never been quantified.

**Methods:**

To compare different training regimens for both attending physicians and first year residents (interns), a prospective study was undertaken to assess knowledge retention six months after an introductory ultrasound course. Eighteen emergency physicians and twelve emergency medicine interns were assessed before and 6 months after an introductory ultrasound course using a standardized, image-based ultrasound test. In addition, the twelve emergency medicine interns were randomized to a group which received additional proctored ultrasound hands-on instruction from qualified faculty or to a control group with no hands-on instruction to determine if proctored exam training impacts ultrasound knowledge. Paired and unpaired estimates of the median shift in test scores between groups were made with the Hodges-Lehmann extension of the Wilcoxon-Mann-Whitney test.

**Results:**

Six months after the introductory course, test scores (out of a 24 point test) were a median of 2.0 (95% CI 1.0 to 3.0) points higher for residents in the control group, 5.0 (95% CI 3.0 to 6.0) points higher for residents in the proctored group, and 2.5 (95% CI 1.0 to 4.0) points higher for the faculty group. Residents randomized to undergo proctored ultrasound examinations exhibited a higher score improvement than their cohorts who were not with a median difference of 3.0 (95% CI 1.0 to 5.0) points.

**Conclusion:**

We conclude that significant improvement in knowledge persists six months after a standard introductory ultrasound course, and incorporating proctored ultrasound training into an emergency ultrasound curriculum may yield even higher knowledge retention.

## Background

Bedside ultrasound (US) has been utilized in the emergency department (ED) setting for nearly two decades [1-6]. With increased use by emergency physicians (EPs) has come mandated training during residency, and many guidelines have been proposed regarding training residents in the use of bedside US. In 1994, the Society for Academic Emergency Medicine (SAEM) proposed a bedside US curriculum consisting of 40 hours of didactic training and 150 proctored US examinations [7]. In 2001, the American College of Emergency Physicians (ACEP) published guidelines describing the scope of emergency US applications as well as recommendations for initial training to include 16 hours of didactic content and a minimum of 150 proctored scans [8].

Despite increasing use of emergency US, training remains inconsistent among EDs nationally [9-11]. Most academic emergency medicine (EM) residency programs have created curricula for ultrasound training ranging from 4 hours [12] to 16 hours [13], however knowledge retention and on-going supplemental training methods have never been investigated. In addition, the need to train EPs who have already completed residency but did not have US training as part of their residency raises additional curriculum questions. Should practicing EPs complete the same didactic curricula as residents? How does their knowledge retention compare?

This study sought to (1) test models of curricula that are typical of those used to train attending emergency physicians and EM residents throughout the country; (2) assess the retention of emergency ultrasound knowledge after an introductory course; and (3) assess the impact of proctored ultrasound training (PUT) on knowledge retention.

## Methods

### Study Design

A cohort design was used to test the hypothesis that scores on an ultrasound test would not change significantly before and after a standard introductory ultrasound course. A randomized controlled study was undertaken in a subset of participants to assess the impact of proctored ultrasound training on test scores. This study was approved by the Institutional Review Board and verbal consent was obtained from all residents participating in the study for inclusion in the randomized part of the protocol. By completing the questionnaire, inclusion in the study was implied for all those not in the proctored group.

### Setting and Population

The study was conducted at two urban university-based emergency departments with an annual census of approximately 70,000 patient visits. Both EDs are staffed by EM board-certified faculty, and are the setting of an EM residency.

Twelve interns in the four-year ACGME-accredited EM residency participated in an emergency US curriculum program during their first two months of residency. None of the twelve interns had received formal ultrasound education as part of their medical school curriculum. Eighteen EM attending faculty from the two affiliated academic EDs completed a 16-hour introductory US course. Of the eighteen faculty, only four reported greater than 10 hours of previous ultrasound training. Twelve attendings had less than four hours of formal ultrasound training and two reported between four and ten hours of training.

### Study Intervention

#### Intern Training

The intern curriculum consisted of 2 separate four-hour blocks of didactic lectures with content focused on the following topics: physics, focused abdominal sonography in trauma (FAST), cardiac, aorta, renal, gallbladder, and pelvic sonography. The didactic content was designed to satisfy the guidelines proposed by ACEP in 2001 and met published guidelines as outlined by Lanoix and Mandavia in previous studies [12,13]. The course was administered over two days, and was taught by the ultrasound director of the program. The ultrasound director is a board-certified EP, fellowship trained in emergency ultrasound, and has been registered as a diagnostic medical sonographer by the American Registry of Diagnostic Medical Sonographers (ARDMS).

#### Intern training- Proctored ultrasounds

To assess the impact of one-on-one proctored ultrasounds on the retention of ultrasound knowledge, six of the twelve interns were randomly selected to receive an additional 90 minute proctored ultrasound examination session with a clinically experienced EP sonographer (ACEP level II credentialed). This consisted entirely of hands on training in performing ultrasounds on live models and was a one-on-one session between the intern and the experienced EP sonographer.

#### Faculty training

The faculty course consisted of 16 hours of lectures, with a printed syllabus covering the following topics: physics, FAST, cardiac, aorta, renal, gallbladder, pelvic sonography. Three of those sixteen hours were supervised hands-on sonography time with live models. The course was administered by faculty from the South Carolina College of Emergency Physicians and is representative of the standard curriculum for continuing medical education courses in emergency ultrasound.

#### Testing

Prior to the start of their courses, interns and faculty each completed the same pretest on emergency US (see Additional file 1). The test was designed to test content from ACEP's curriculum guidelines, and each question was image-based. There were 24 questions, four on each of the six applications representative of the major areas of emergency US (FAST, cardiac, aorta, renal, gallbladder, and pelvic sonography). Test items required recognition of positive, negative, and nondiagnostic or technically limited study (TLS) images. There were seven positive scans to identify, seven negative scans, six nondiagnostic scans and four general ultrasound anatomy recognition scans. Each correct answer was given a score of 1 point out of a total possible score of 24.

Six months after the completion of their respective introductory courses, all interns and faculty completed a post-test consisting of the exact same questions as the pre-test. During the intervening six month period, neither faculty nor residents were exposed to any further ultrasound focused didactic curricula. In both cohorts, none of the eighteen attendings and none of the twelve residents took any additional outside courses in emergency ultrasound. In addition, emergency ultrasound was not part of the resident training program or the standard workflow at either participating hospital, so there was minimal exposure to emergency ultrasound on a clinical basis during the six month study period.

### Measurement and outcomes

Pre- and post-test scores were recorded for all faculty and residents undergoing the introductory course. The two primary outcomes were the change in test scores for residents and faculty (pre-test vs. post-test), and the difference in score improvement between the residents randomized to undergo proctored ultrasound examinations compared to those who were not. A secondary outcome was a comparison of the change in pre- and post-training scores for questions with positive, negative, or nondiagnostic scans in the faculty group.

### Data Analysis

Data was recorded in an Excel (Microsoft Office XP, Redmond, WA) spreadsheet, then transferred to SPSS (SPSS V.14, Chicago, IL) and Statxact (Statxact 3, Cytel Software, Cambridge, MA) databases for graphic and quantitative analyses. Given the relatively small datasets, all univariate comparisons were performed with exact statistics using Statxact. The Hodges-Lehmann extension of the Wilcoxon-Mann-Whitney test was used to estimate the median shift parameter for comparisons between two samples. Comparisons were paired or unpaired, as appropriate. For the comparison of three related samples, the Friedman test was used. Power calculation was not performed prior to the study.

For the intern trainees, the pre-test control (interns randomized to no PUT) and treatment (interns randomized to PUT) group scores were compared to assess the difference in the pre-test abilities of each group. Pre- and post-training scores were compared using paired test assessments for each group. The difference in pre- and post-test scores for both treatment and control groups were then compared.

The faculty training group had a paired test assessment performed to compare the pre- and post-training test scores. The change in pre- and post-training test scores for the positive, negative, or nondiagnostic exam questions were compared using the Friedman test.

## Results

### Resident Training

**Figure 1:**
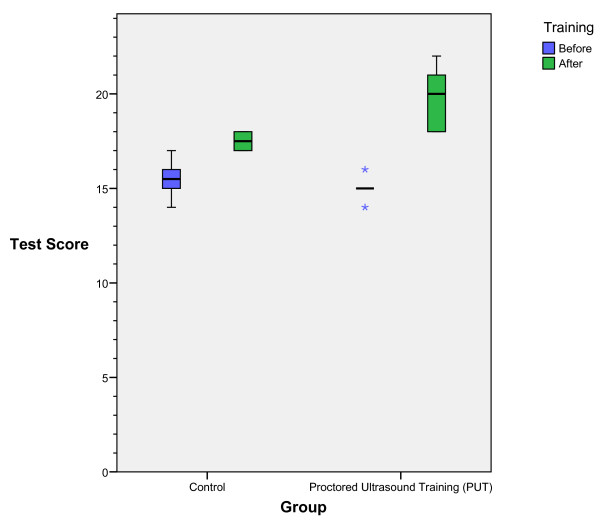
Intern controls and PUT group pre and post-training test scores. The central line is the median, the box represents the interquartile range (the 25th to 75th percentile), the whiskers extend to the largest and smallest values within 1.5 box lengths, and circles and asterisks represent outliers within or greater than 3 box lengths from the box edge, respectively.

Twelve interns enrolled in the study and all completed the pre-test, curriculum, and post-test. None had prior formal emergency bedside US training and none had any additional non-study mandated ultrasound training during the six-month period between tests. The median difference in the pre-test scores between the control and PUT groups was 0.5 (95% CI -1.0 to 2.0). Comparing pre- and post-test scores in the control group, the median improvement in score was 2.0 (95% CI 1.0 to 3.0). Improvement was found in the PUT group as well with a median value of 5.0 (95% CI 3.0 to 6.0). The improvement in scores between the pre-test and post-test for the PUT group was greater than that of the control with a median difference in improvement of 3.0 (95% CI 1.0 to 5.0) (see figure 1).

### Faculty training

**Figure 2:**
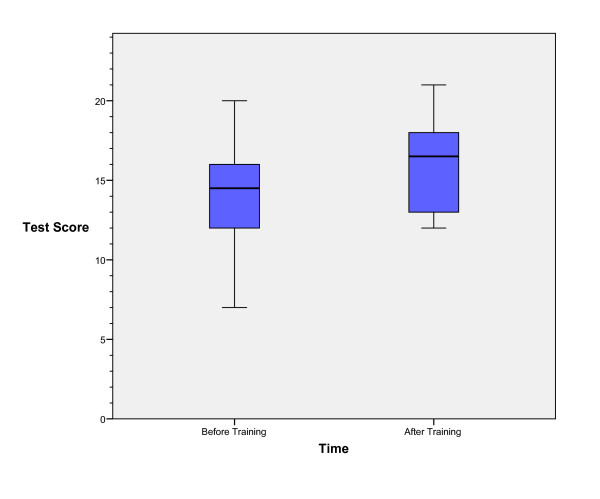
Faculty pre and post-training test scores.

**Figure 3:**
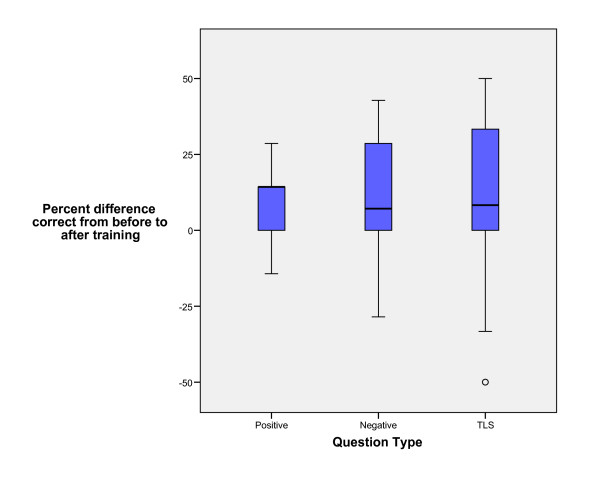
Faculty test score improvement by question category (true positive image, true negative image, non-diagnostic image or technically limited study (TLS)).

Eighteen emergency attending physicians (76% with no previous formal emergency bedside US training) completed the introductory pre-test, curriculum, and post-test. None of the faculty had any additional non-study mandated ultrasound training during the six-month period post training. The median overall test score improved 2.5 (95% CI 1.0 to 4.0) from before the course to 6 months after (see figure 2). Comparing the test results for the positive, negative or TLS/nondiagnostic questions, there was no significant difference in the improvement from before training to 6 months after (p = 0.86) (see figure 3.

## Discussion

With the introduction of new technologies, all physicians are required to undergo continuing education in order to familiarize themselves with a changing medical landscape. This is being done in surgery, cardiology, internal medicine, obstetrics and almost every specialty profession as new technologies change the practice of that specialty. Emergency medicine is no different and bedside US is only one of many new technologies where proficiency is required of EPs. The dilemma is that the acquisition of new technical skills has not traditionally been quantified. Residency curricula usually involves didactic lectures introducing topics and then "real-life, real-time" application when working in the emergency department. Faculty gauge residents' knowledge retention by how they perform clinically and how their patients do. With bedside ultrasound, the ability to evaluate each resident's specificity and sensitivity for their ultrasound diagnostic accuracy suggests the need to have a more rigorous quality assurance process in addition to continuing education to maintain diagnostic accuracy. This becomes even more important when we look at how we are training practicing EPs who have completed residency and did not have formal bedside US training.

There has been much evidence in the literature to show that with focused US training, emergency medicine residents and faculty are able to make diagnoses accurately [1,2,4,9-14]. This study supports the notion that with focused didactic and hands-on experience, interpretative accuracy improves and that the level of improvement is sustained over 6 months. More importantly, however, it suggests that adding one proctored hands-on session had a significant impact in image recognition and retention of knowledge. While more labor intensive than group courses, PUT adds significant value to traditional didactic teaching. In addition to improving image recognition, residents in the PUT group subjectively described improved comfort with the ultrasound machine and increased frequency of use in the subjective comments section of the post-test. Comments from all six PUT interns ranged from "This will definitely help me use the ultrasound machine more frequently" to "I feel much more confident in my ultrasound abilities". The six control group interns and faculty participants had subjective comments of which the following is more representative: "This course helped familiarize me with ultrasound but I am still not sure I am confident in my abilities to make diagnoses independently." This implies that PUT may not only have more impact on image recognition, but also may be a more important component in curriculum development for improving physician confidence.

This study is limited by its sample size and by the fact that the faculty and residents are from a single residency program. It also did not assess image acquisition skills over time, only diagnostic ability and image recognition. Obviously, in order to be performed proficiently both acquisition and interpretation of ultrasound images need to be accurate. However, this study does suggest that proctored exam teaching may be beneficial for emergency medicine resident training. If PUT could improve knowledge retention for faculty and in addition positively impact their comfort level with the technology, PUT may be worth the additional investment. Further study should include PUT as part of the training module for both faculty and residents and track image accuracy (acquisition and interpretation) over time.

## Conclusion

This study suggests US knowledge is retained for at least 6 months both after a 16-hr and an 8-hr course, even in the absence of formalized continuing US educational interventions. The addition of proctored exams to an ultrasound curriculum is demonstrated to be of high utility leading to an even greater sustained improvement in image interpretative skills. Further study is needed to quantify the optimal time spent in didactic training and proctored exams, but the resources spent on proctored exams seem to be well spent with sustainable results.

## Competing interests

The author(s) declare that they have no competing interests.

## Authors' contributions

VN, BN, AS and HC participated in the design of this study and carried out the design, coordination and test administration. VN and BN drafted the manuscript and KM performed the statistical analysis. All authors read, revised and approved of the final manuscript

## Pre-publication history

The pre-publication history for this paper can be accessed here:



## Supplementary Material

Additional file 1 Ultrasound Image Recognition Test. Copy of test used in the study with questions and images. Click here for file
