# Supplementary material for: Assessment of knowledge retention and the value of proctored ultrasound exams after the introduction of an emergency ultrasound curriculum
Source: BMC Med Educ. 2007 Oct 30;7:40. doi: 10.1186/1472-6920-7-40 (PMC2223143; doi:10.1186/1472-6920-7-40)

# Ultrasound Pretest

Name:_____________________________________________

Degree(s):_________________________

Level of training:

EM-1 EM-2 EM-3 EM-4 Attending (year graduated residency:___________)

Years of residency training:

1. 4 Other:______________

Level of prior training in ED ultrasound:

None 1-4 hours 5-10 hours >10 hours

Obtained ED ultrasound experience via:

Residency training ultrasound course Other:_________________

Indicate (circle) which of the following areas of ED ultrasound you have had prior experience with:

Trauma

AAA

Gallbladder disease

Renal disease
Cardiac ultrasound

Obstetrical ultrasound

1. A 45 year-old woman presents with nausea, vomiting, and right upper quadrant pain associated with meals. She notes tenderness as you obtain the image below. What is the likelihood that she has acute cholecystitis?

a. 0-10%

b. 11-40%

c. 41-75%

d. 76-90%

e. 91-100%


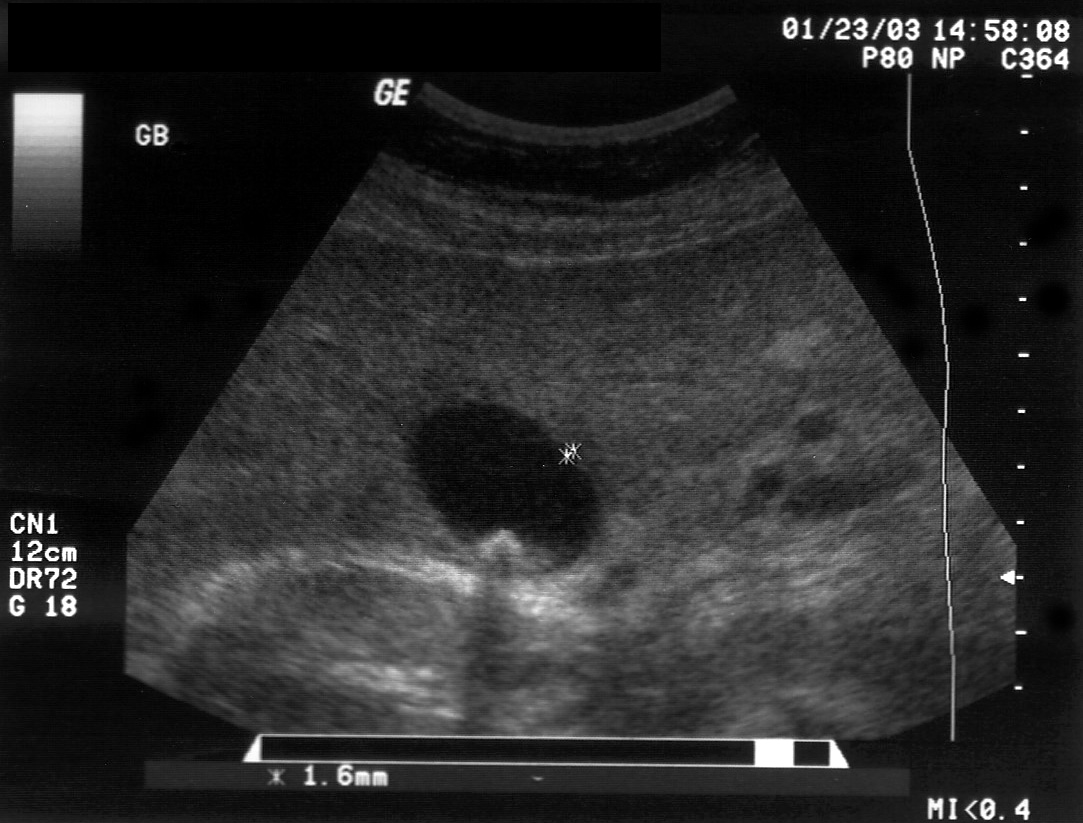


2. An elderly man presents to the ED with flank pain and hematuria. You obtain the image below and interpret it as:

a. positive for AAA

b. negative for AAA

c. an incomplete study of the aorta

d. hydronephrosis


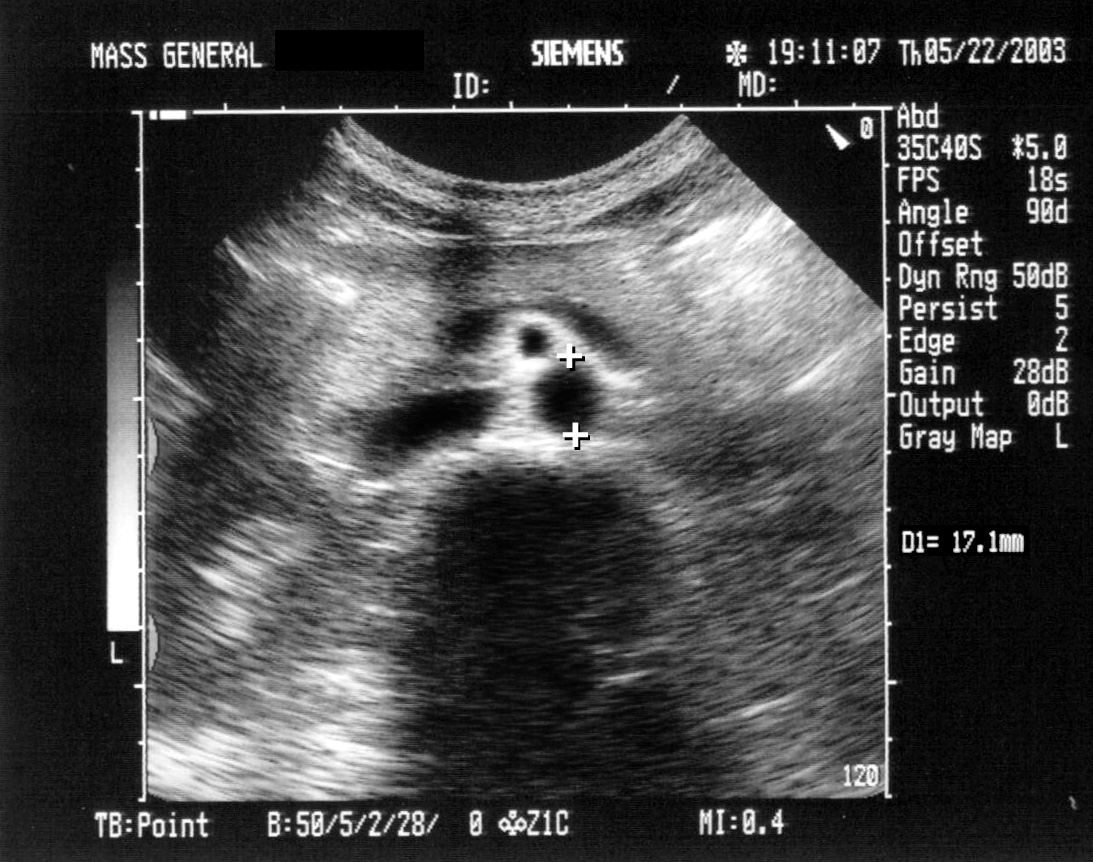


3. What structure is indicated by the arrow in the above image?

a. Splenic vein

b. Inferior vena cava

c. Lumbar shadow

d. Superior mesenteric artery

4. A 40 year-old man presents complaining of flank pain. You obtain the following renal ultrasound. It demonstrates:

a. Hydronephrosis

b. A normal kidney

c. Subcapsular hematoma

d. Simple renal cyst


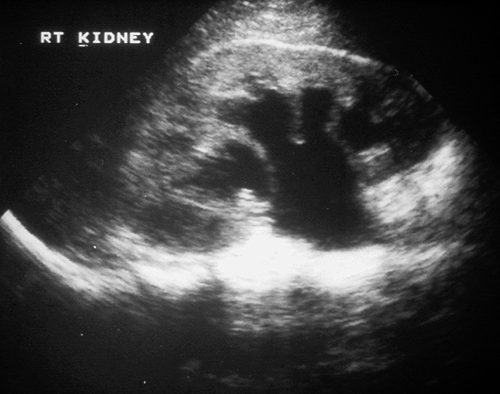


5. A 23-year old woman with a positive home pregnancy test presents with vaginal bleeding. You perform a bedside transabdominal ultrasound and obtain the transverse image of the bladder and uterus below. You interpret it as:

a. No definitive IUP

b. IUP present

c. Additional images are needed to assess for the presence of an IUP


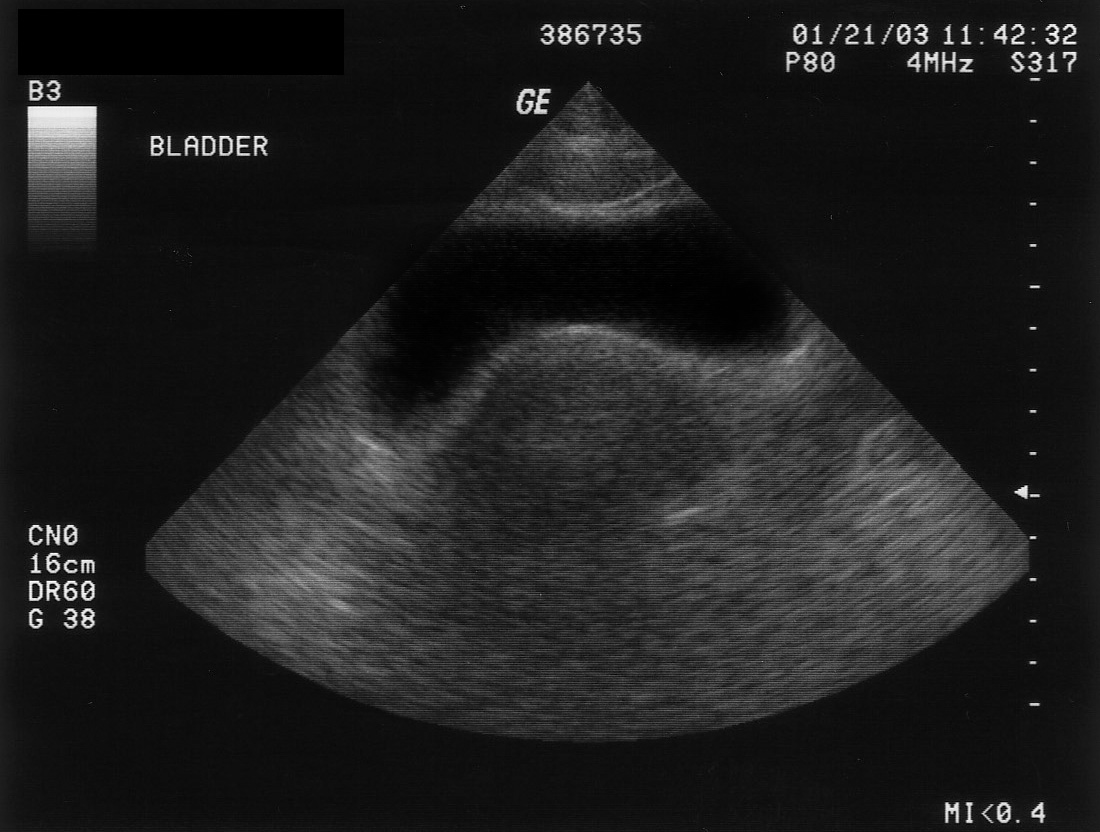


6. After placing a Foley catheter in an elderly man complaining of urinary retention, you obtain the following ultrasound, and note:

a. Hydronephrosis (mild)

b. A normal kidney

c. Ascites

d. Simple renal cyst


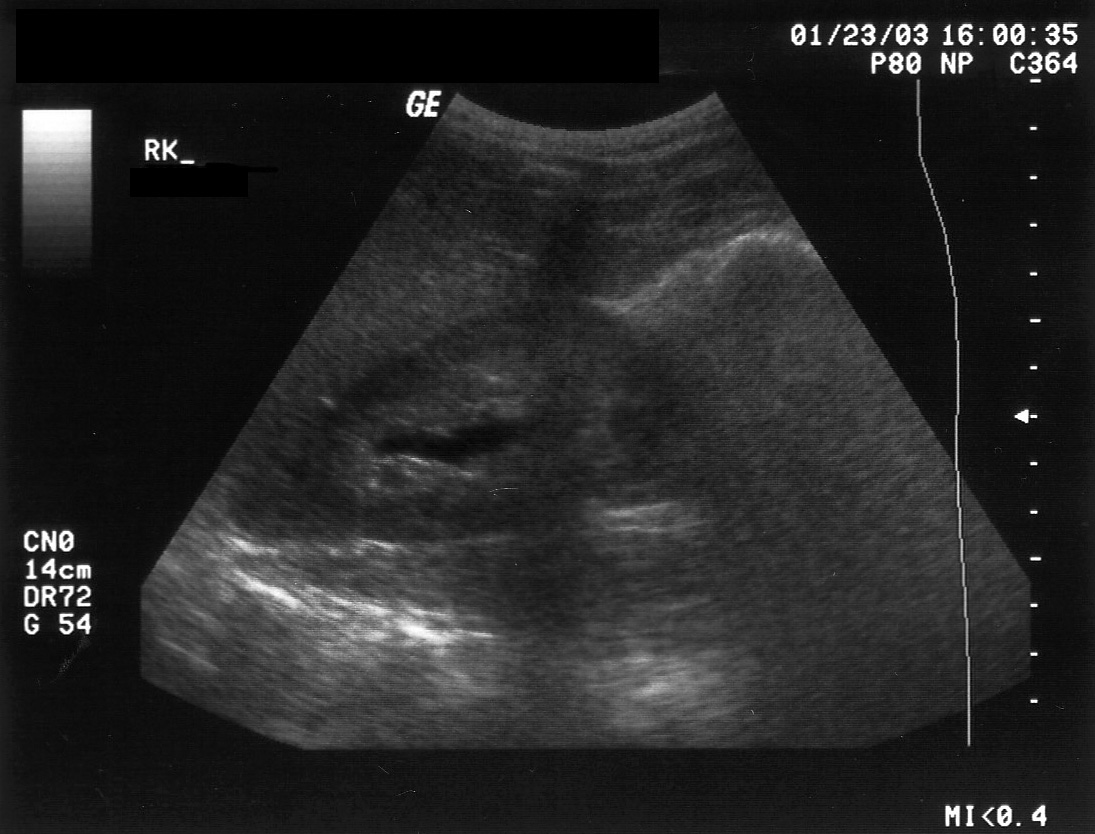


7. An elderly man presents to the ED with flank pain and hematuria. You obtain the image of the aorta below and interpret it as:

a. positive for AAA

b. negative for AAA

c. an incomplete study

d. hydronephrosis


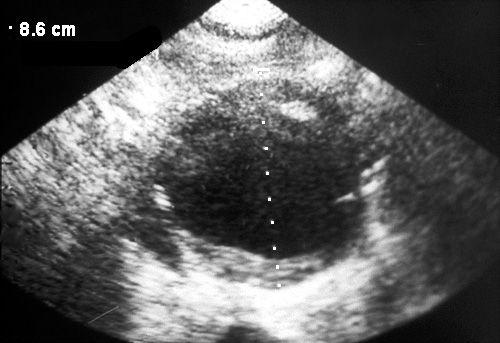


8. An 80 year-old woman complains of right flank pain. You obtain a longitudinal view of the kidney which demonstrates:

a. Hydronephrosis

b. No hydronephrosis

c. Ascites

c. AAA


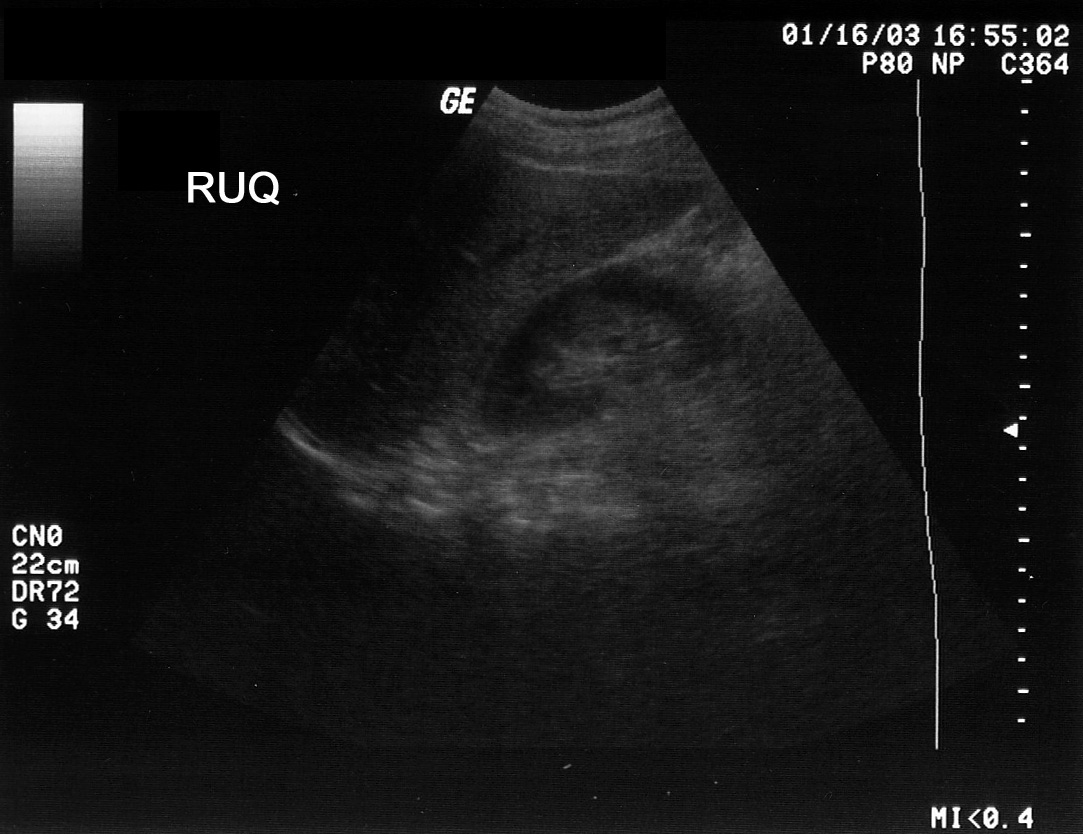


9. A young girl sustained left –sided trauma by hitting her abdomen on her handlebars. The ultrasound of the splenorenal space below should be interpreted as:

a. Positive for free intraperitoneal fluid in the splenorenal space

b. Negative for free intraperitoneal fluid in the splenorenal space

c. Nondiagnostic for free fluid in the splenorenal space


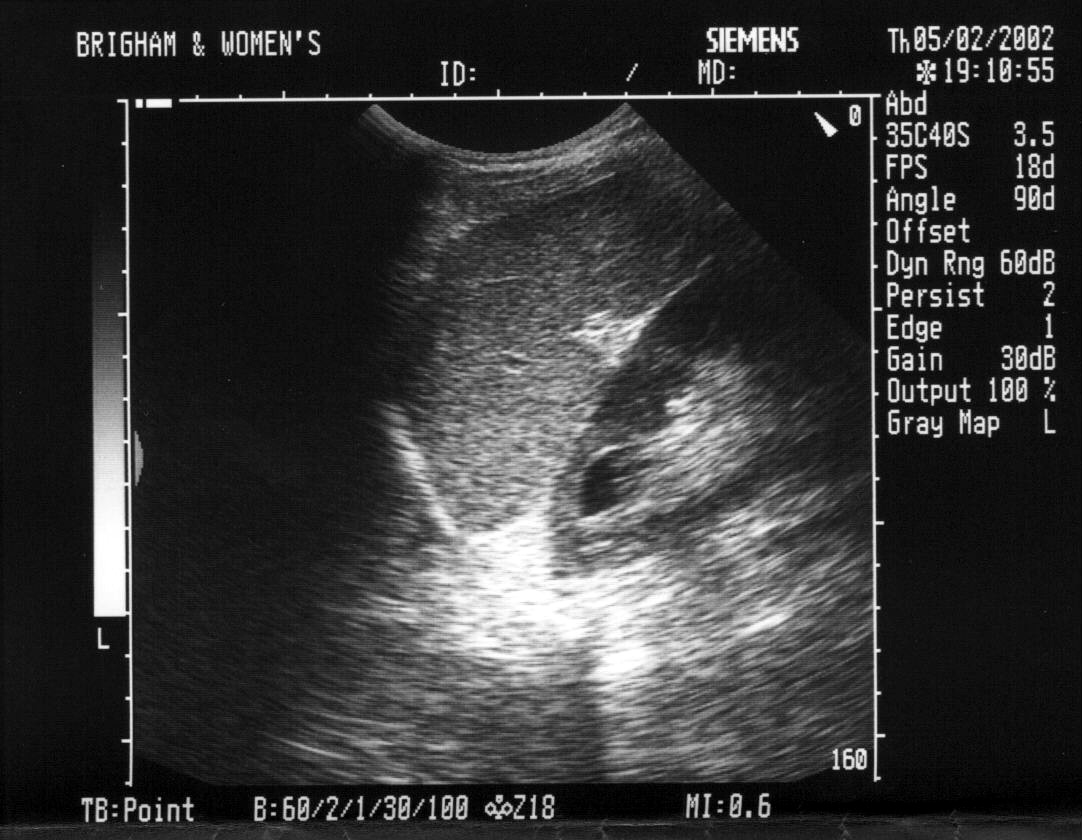


10. You obtain a transverse view of the bladder in a 22 year-old male patient with hypotension after blunt abdominal trauma. This scan is:

a. Positive for free fluid in the pouch of Douglas

b. Negative for free fluid in the pouch of Douglas

c. Nondiagnostic for free fluid


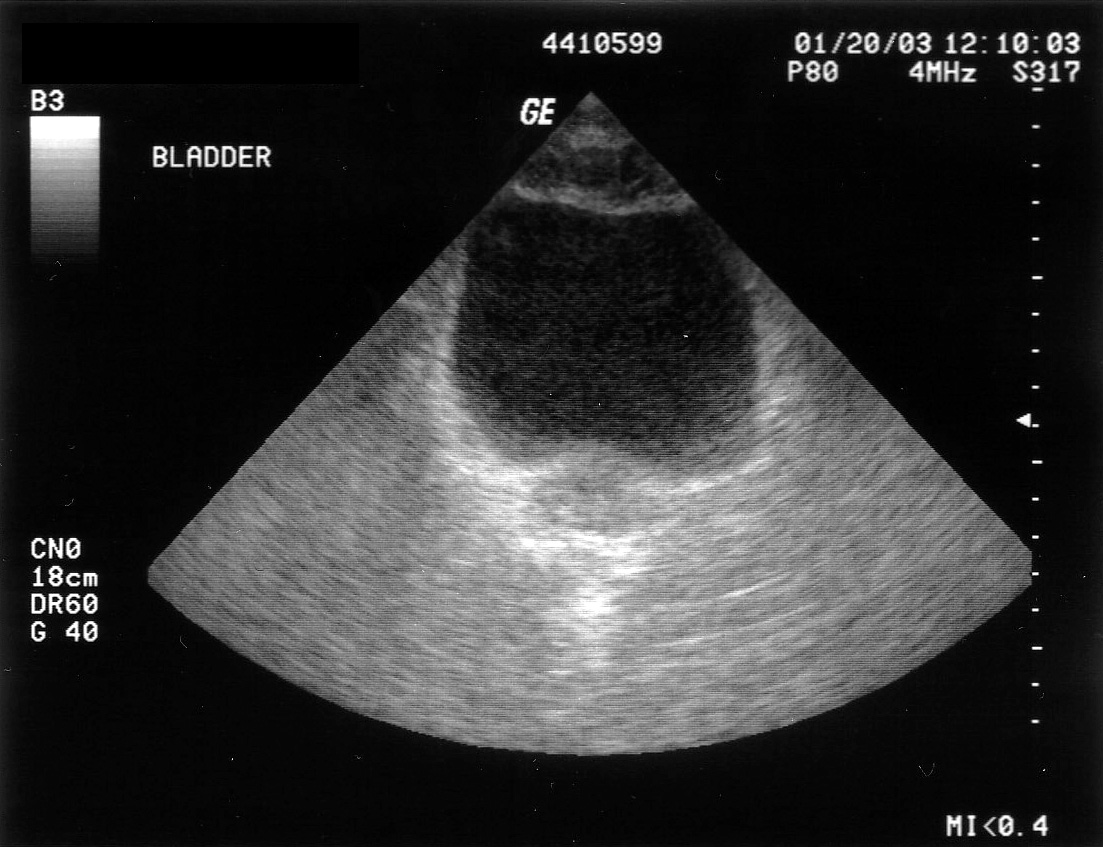


11. As part of a FAST exam, you obtain the following image in the right upper quadrant of a blunt trauma patient. It should be interpreted as:

a. Adequate view of Morrison’s pouch, free intraperitoneal fluid

b. Inadequate view of Morrison’s pouch, free intraperitoneal fluid

c. Adequate view of Morrison’s pouch, pneumothorax

d. Inadequate view of Morrison’s pouch, pleural effusion


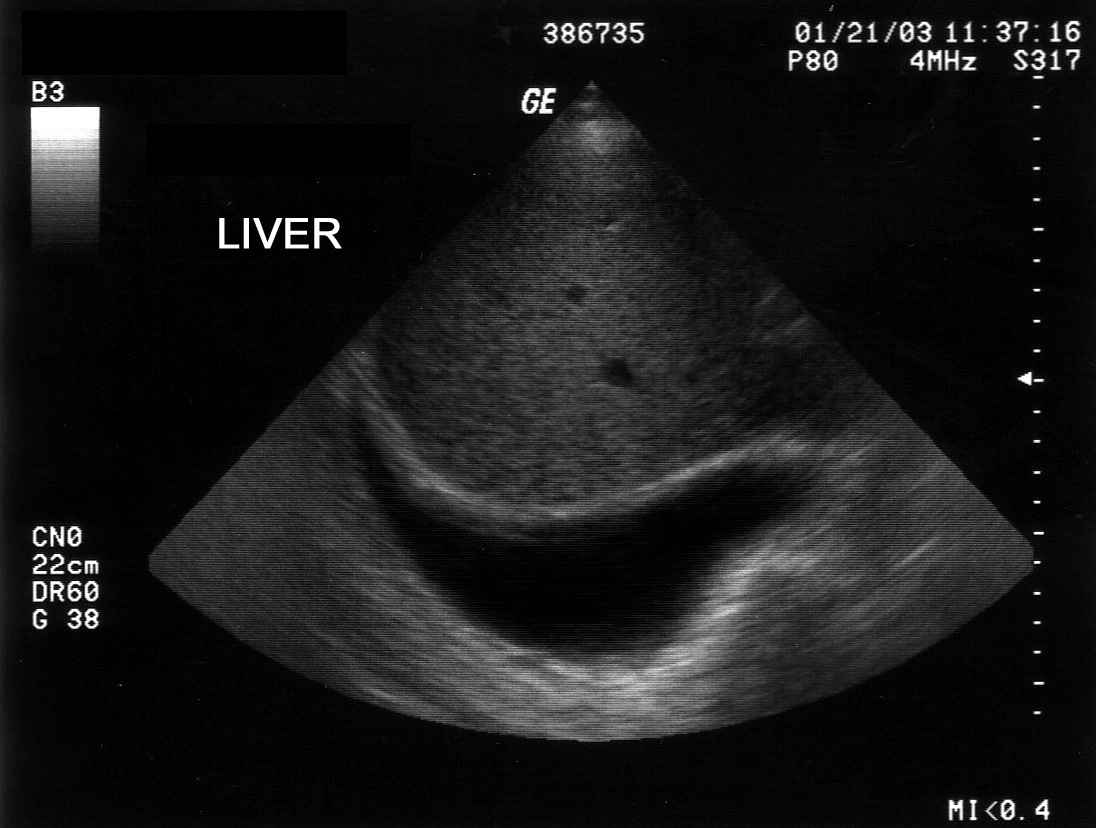


12. An elderly man presents to the ED with fever and abdominal pain. You obtain the following image of the gallbladder, common bile duct, and portal vein. Which of the following are evident on the scan below?

a. Gallstones

b. Pericholecystic fluid

c. Thickened gallbladder wall

d. Dilated common bile duct

e. None of the above


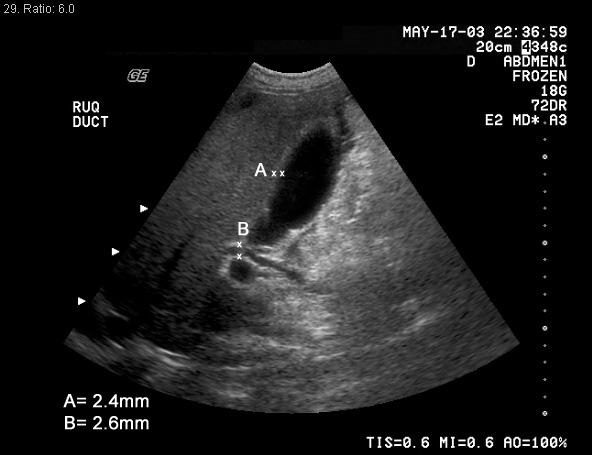


13. What structure is indicated by the arrow?

a. Common bile duct

b. Inferior vena cava

c. Portal vein

d. Gallstone

14. A young man sustained a stab wound to the chest. You perform a cardiac exam, and the subxyphoid view is below. You diagnose:

a. Pericardial effusion

b. No pericardial effusion

c. A nondiagnostic exam


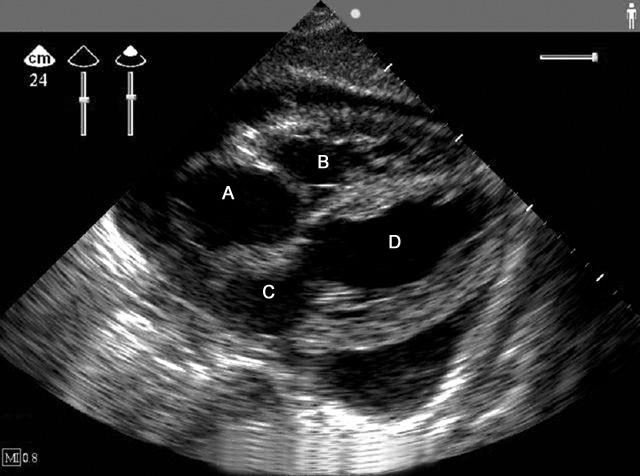


15. Which label correctly identifies the chamber that would collapse earliest during early tamponade?

a. A

b. B

c. C

d. D

16. A 32-year old woman with a positive home pregnancy test presents with vaginal bleeding. You perform a bedside transabdominal ultrasound and obtain the longitudinal image below. You interpret it as:

a. No definitive IUP

b. IUP present

c. Ruptured ectopic pregnancy


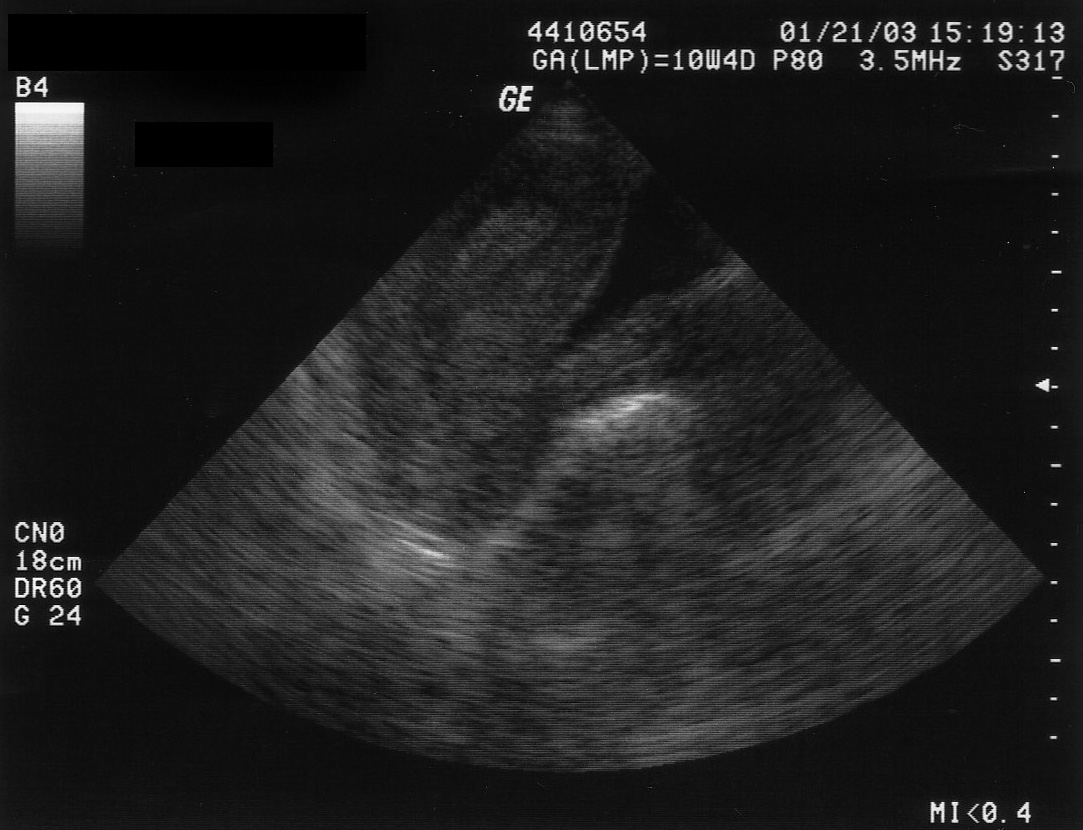


17. What structure is indicated by the arrow?

a. Endometrial stripe

b. Myometrium

c. Free fluid/blood

d. Bladder

18. A patient presents in PEA arrest. Bedside ultrasound demonstrates the image below (with cardiac motion present). Based on this image, you could:

a. Perform pericardiocentesis

b. Not perform pericardiocentesis

c. Attempt another view, as this is not adequate to assess pericardial effusion


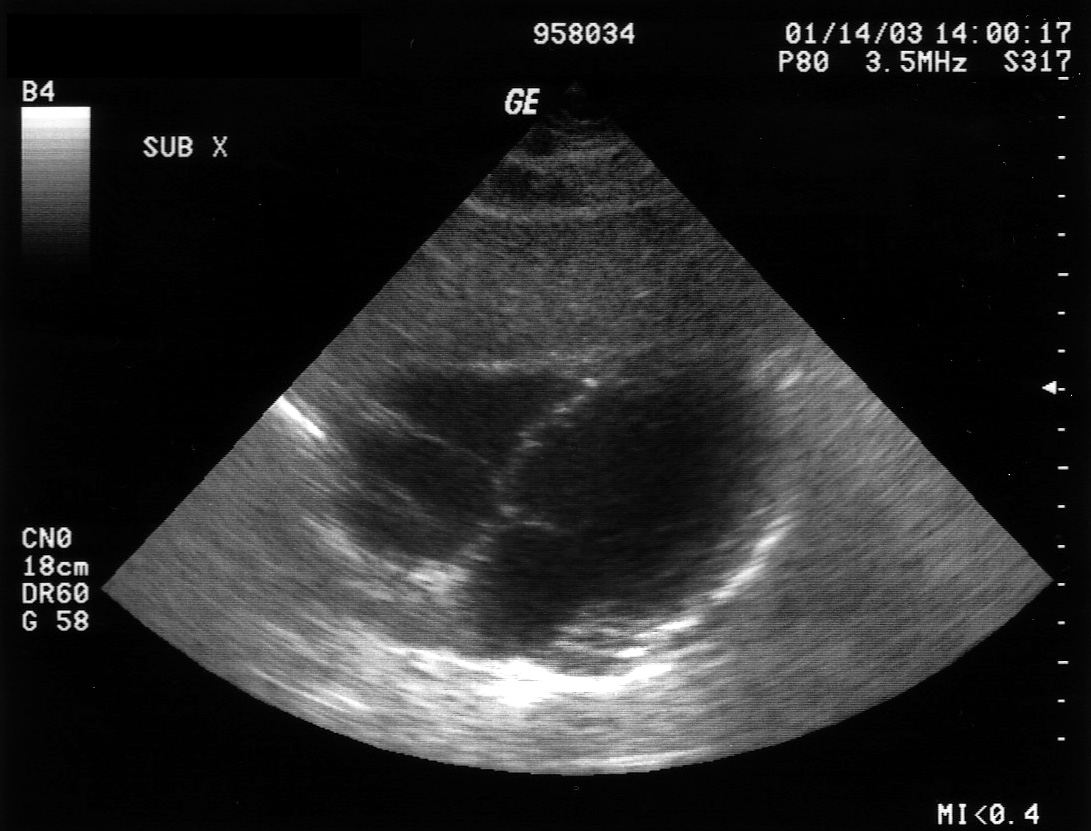


19. You obtain the following image of the liver and kidney in the right upper quadrant of a patient complaining of hematuria. It should be interpreted as:

a. Hydronephrosis

b. No hydronephrosis

c. Inadequate view of the kidney


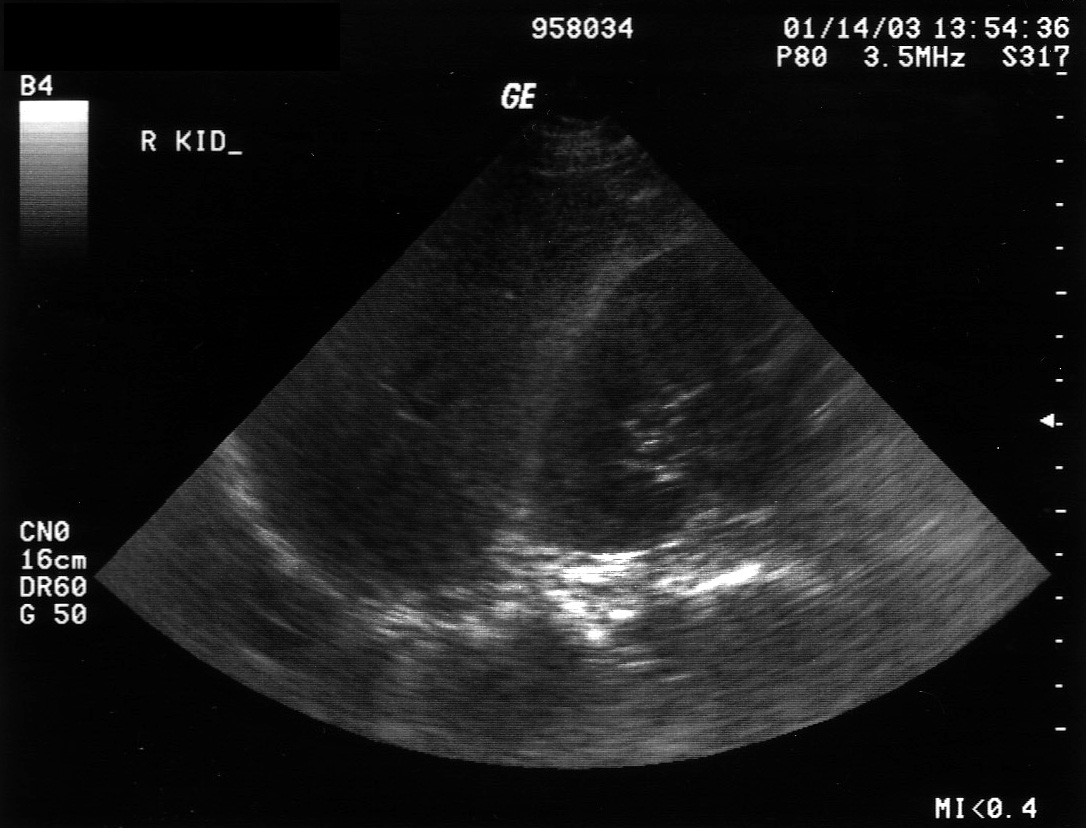


20. As part of a FAST exam, you obtain the following image in the right upper quadrant of a blunt trauma patient. It should be interpreted as:

a. An incomplete view of Morrison’s pouch

b. Positive for free intraperitoneal fluid

c. Negative for free intraperitoneal fluid


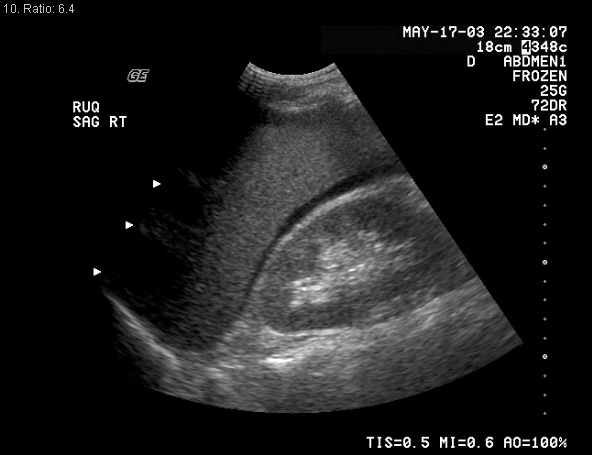


21. A woman presents with vaginal bleeding, an unclear menstrual history and a positive home pregnancy test. Based on the image of the uterus below, you:

a. Confirm IUP

b. Suspect ectopic pregnancy

c. Cannot determine the presence of IUP with this image


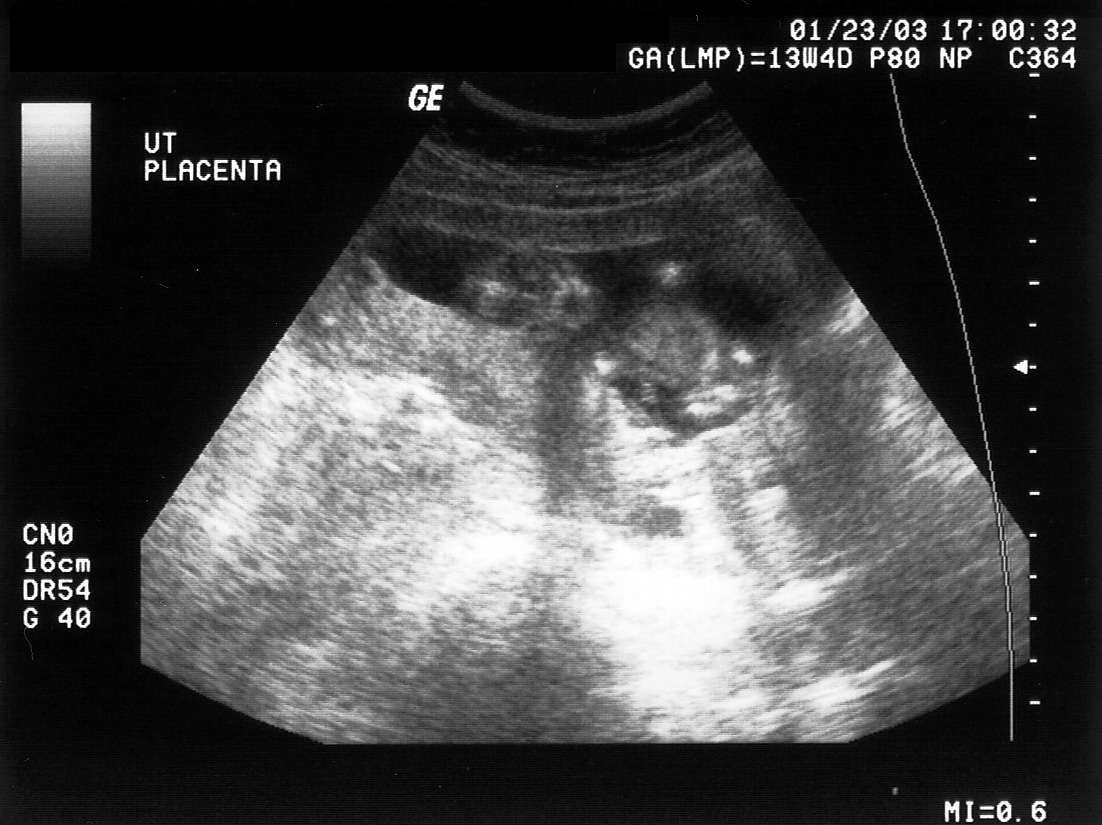


22. Your colleague shows you the image below, obtained in a patient with RUQ pain. Based on the transverse image of the gallbladder below, you:

a. Rule out gallstones

b. Confirm gallstones

c. Tactfully ask your colleague to complete this limited study with additional images


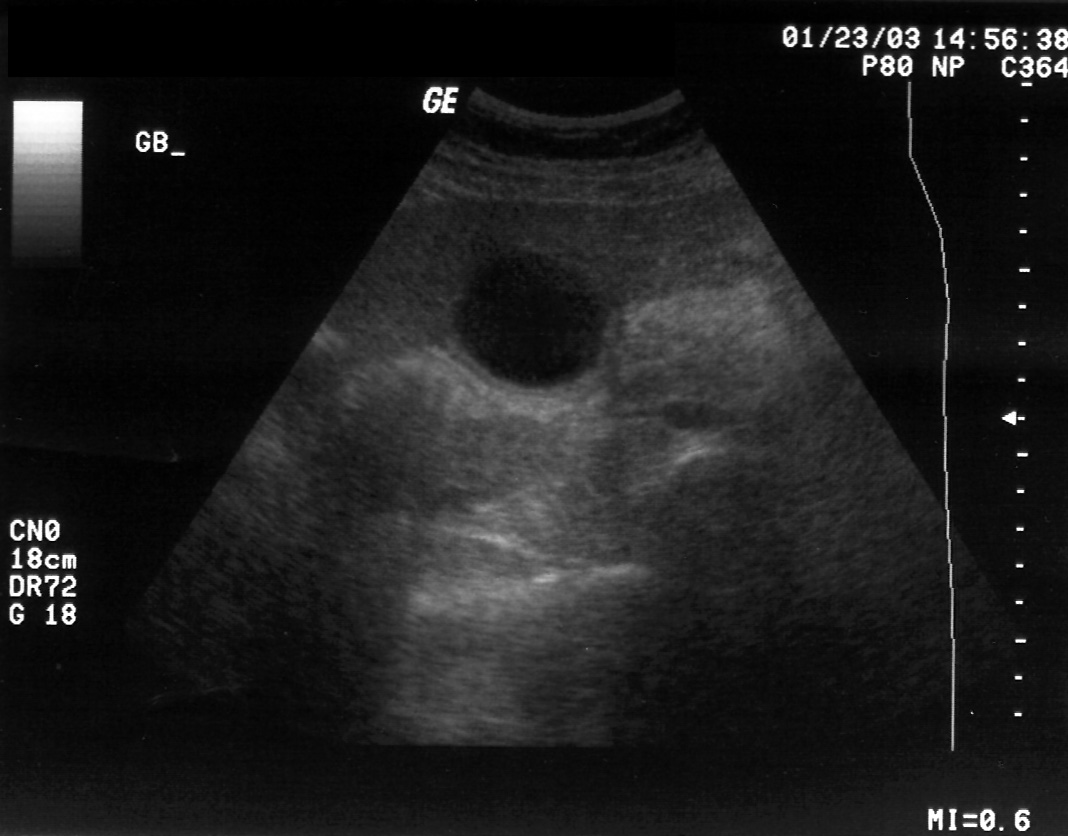


23. You perform an aortic ultrasound to assess for the presence of abdominal aortic aneurysm. What conclusions can you draw from this image?

a. The diameter of the vessel is normal at this level

b. There is an aneurysm at this level

c. There is a large intramural thrombus present


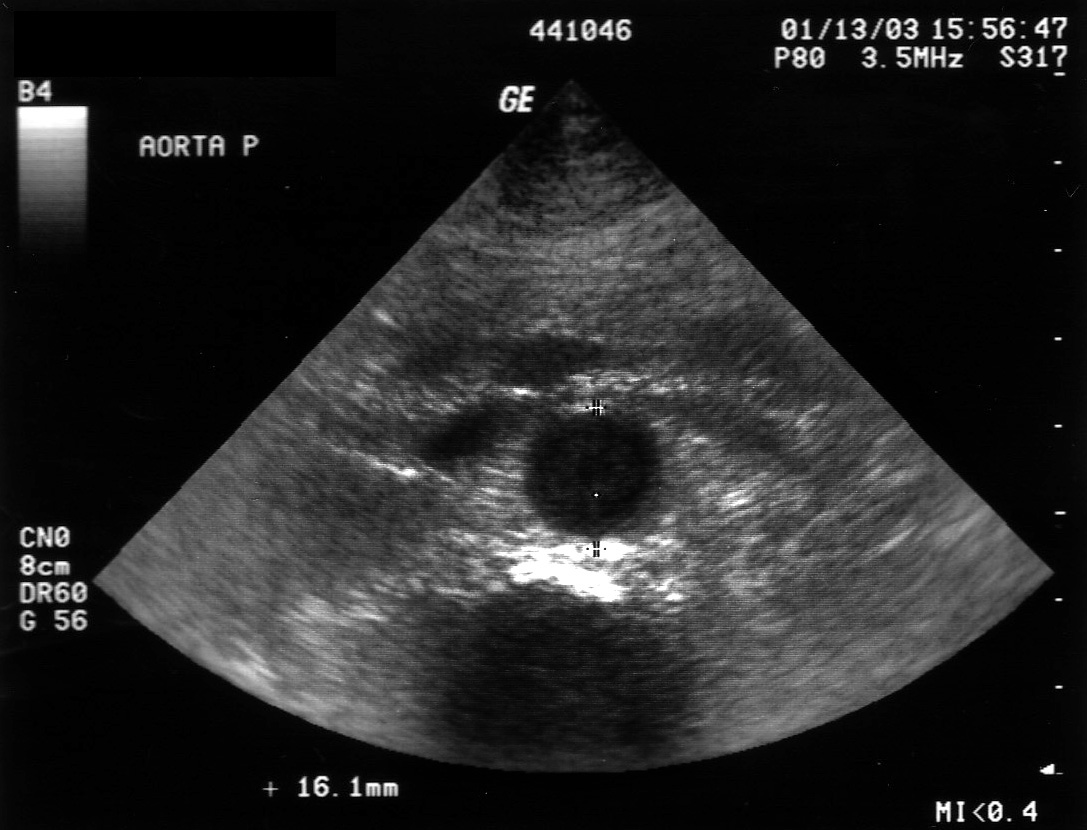


24. A patient presents with hypotension after a stab wound to the chest. Bedside ultrasound demonstrates the image below (with cardiac motion present). Based on this image, you could:

a. Perform pericardiocentesis

b. Not perform pericardiocentesis

c. Attempt another view, as this is not adequate to assess pericardial effusion


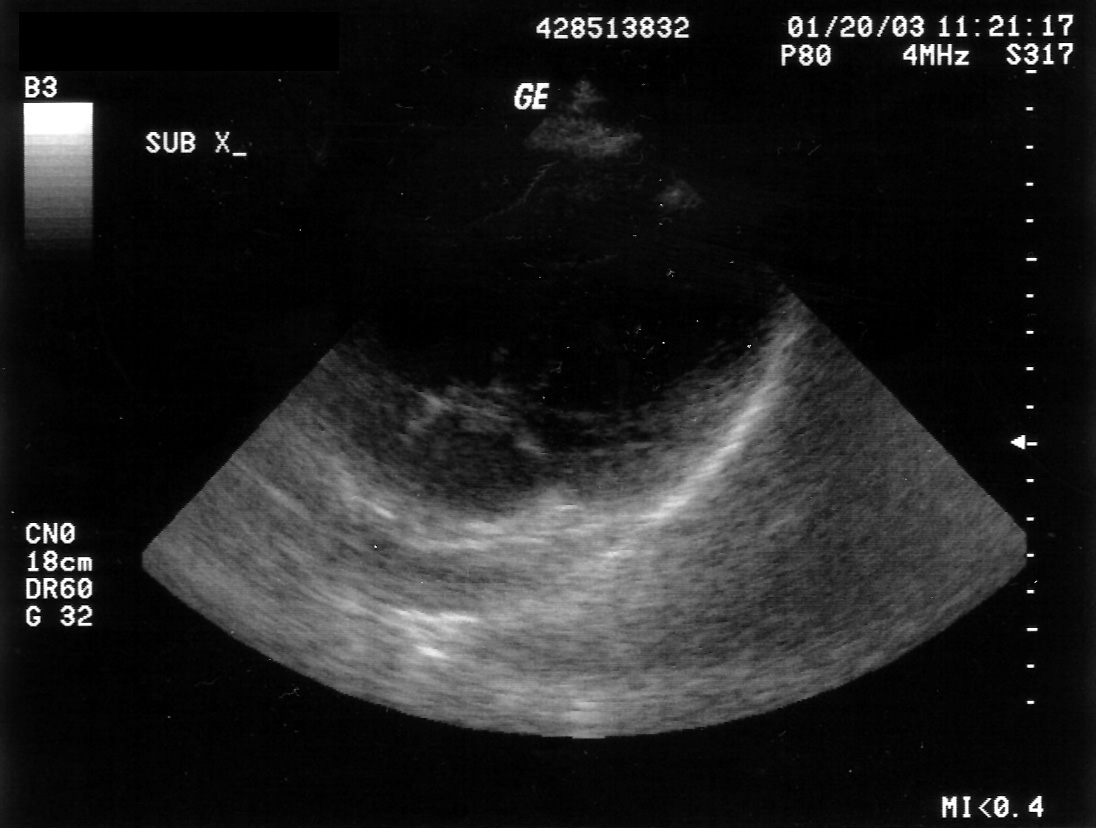

Supplement: Additional file 1 — Ultrasound Image Recognition Test. Copy of test used in the study with questions and images. [file 1472-6920-7-40-S1.doc]
